# Predicting effect of anti-PD-1/PD-L1 inhibitors therapy for hepatocellular carcinoma by detecting plasma metabolite based on UHPLC-MS

**DOI:** 10.3389/fimmu.2024.1370771

**Published:** 2024-04-18

**Authors:** Botong Liu, Jinyu Shi, Rui Su, Ran Zheng, Fan Xing, Yuan Zhang, Nanya Wang, Huanwen Chen, Shouhua Feng

**Affiliations:** ^1^ State Key Laboratory of Inorganic Synthesis and Preparative Chemistry, College of Chemistry, Jilin University, Changchun, China; ^2^ The Cancer Center, The First Hospital of Jilin University, Changchun, China; ^3^ School of Pharmacy, Jiangxi University of Chinese Medicine, Nanchang, China

**Keywords:** anti-PD-1/PD-L1 inhibitors therapy, hepatocellular carcinoma, UHPLC-MS, plasma metabolite, prognosis

## Abstract

**Introduction:**

Anti-PD-1/PD-L1 inhibitors therapy has become a promising treatment for hepatocellular carcinoma (HCC), while the therapeutic efficacy varies significantly among effects for individual patients are significant difference. Unfortunately, specific predictive biomarkers indicating the degree of benefit for patients and thus guiding the selection of suitable candidates for immune therapy remain elusive.no specific predictive biomarkers are available indicating the degree of benefit for patients and thus screening the preferred population suitable for the immune therapy.

**Methods:**

Ultra-high-pressure liquid chromatography-mass spectrometry (UHPLC-MS) considered is an important method for analyzing biological samples, since it has the advantages of high rapid, high sensitivity, and high specificity. Ultra-high-pressure liquid chromatography-mass spectrometry (UHPLC-MS) has emerged as a pivotal method for analyzing biological samples due to its inherent advantages of rapidity, sensitivity, and specificity. In this study, potential metabolite biomarkers that can predict the therapeutic effect of HCC patients receiving immune therapy were identified by UHPLC-MS.

**Results:**

A partial least-squares discriminant analysis (PLS-DA) model was established using 14 glycerophospholipid metabolites mentioned above, and good prediction parameters (R2 = 0.823, Q2 = 0.615, prediction accuracy = 0.880 and p < 0.001) were obtained. The relative abundance of glycerophospholipid metabolite ions is closely related to the survival benefit of HCC patients who received immune therapy.

**Discussion:**

This study reveals that glycerophospholipid metabolites play a crucial role in predicting the efficacy of immune therapy for HCC.

## 1 Background

Hepatocellular carcinoma (HCC) is a malignant tumor with a high incidence and mortality worldwide (1). Multicenter, randomized clinical trials, such as IMbrave 150 and Orient 32, have been demonstrated the effectiveness of programmed cell death protein 1/programmed death ligand 1 (PD-1/PD-L1) inhibitor based anti-PD-1/PD-L1 therapy in HCC patients (2–6). Anti-PD-1/PD-L1 therapy was proven to increase the tumor response and prolong the survival in patients with advanced HCC. However, it still faces challenges. Currently, the objective response rate (ORR) of anti-PD-1/PD-L1 therapy for HCC was only 17%-30% (2–8). Long-term survival benefits are achieved for some patients, while primary resistance or hyperprogression in very short time occur in some patients after receiving anti-PD-1/PD-L1 therapy. In addition, adverse effect also limits its clinical application treating HCC. Therefore, there is extremely urgent to explore biomarkers predicting the treatment response of immune therapy in the early time.

Biomarkers such as PD-L1 expression in tumor tissues, tumor mutation burden (TMB), mismatch repair gene (MMR), and microsatellite instability (MSI) (9–21) could predict the prognosis of HCC patients receiving anti-PD-1/PD-L1 therapy. However, all these biomarkers are detected by tumor tissue and are not easily obtained. Furthermore, the intertumoral heterogeneity may significantly affect the results. Currently, there are no biomarkers that can simply and accurately predict the prognosis of HCC patients receiving anti-PD-1/PD-L1 therapy in clinical practice. Therefore, it is critical to explore suitable biomarkers which accurately predict the prognosis of HCC patients receiving anti-PD-1/PD-L1 therapy, especially screen the advantage population for anti-PD-1/PD-L1 therapy before treatment, assist clinicians in making treatment decisions, and improve the prognosis of HCC patients.

UHPLC-MS allows simultaneous detection of several metabolites with high quality data (22), and thus is well suitable for discovering biomarkers (23).Currently, some liver diseases including diagnosis of HCC (24–27), the prognosis of HCC (28–30), differentiation of HCC from other liver disease (31–34) have been studied by applying metabolomics. To date, many metabolites were reported as potential biomarkers for liver disease. However, no biomarkers predicting the therapeutic effect of anti-PD-1/PD-L1 therapy for HCC were reported.

Herein, metabolic profiling data were obtained from UHPLC-MS to elucidate the abnormal metabolism associated with HCC patients. A total of 59 participants were enrolled to explore metabolic features and metabolic pathways related to HCC patients. In addition, a two-step partial least-squares discriminant analysis (PLS-DA) model analysis strategy including all metabolites and characteristic metabolites was established to discover and validate new biomarkers set to predict the efficacy of immune therapy for HCC. Using this strategy, six potential biomarkers were found. In addition, we found that the alteration of PE (36:4) in the progression-free survival (PFS) and overall survival (OS) of patients was significant.

## 2 Methods

### 2.1 Reagents and apparatus

Acetonitrile (HPLC grade) was purchased from Merck KGaA (Darmstadt, Germany). Formic acid (LC/MS grade) was purchased from Energy Chemical (Shanghai, China). Ammonium Formate (Optima LC/MS grade) was purchased from Fisher Scientific (Fair Lawn, USA). Ultrapure water was purchased from Watsons (Guangzhou, China).

### 2.2 Patients

Consecutive HCC patients who received anti-PD-1/PD-L1 therapy from January 2020 to December 2020 at the First Hospital of Jilin University (Changchun City, Jilin Province, China) were identified. A total of 65 patients were initially selected, and 59 patients were finally chosen for the study (**Supplementary Figure S1**). The Institutional Review Board approved the present study of the First Hospital of Jilin University. Written, informed consent for their serum samples to be used for biomedical research was obtained from all enrolled patients. This study was conducted according to the Declaration of Helsinki and the Ethical Guidelines for Clinical Studies.

Inclusion criteria includes: 1) the diagnosis of HCC was confirmed by pathological examination or clinical diagnosis, 2) locally advanced or distant metastases and after multidisciplinary consultation, it was determined that local treatment was not suitable 3) received the anti-PD-1/PD-L1 therapy and baseline reference serum samples were collected, 4) underwent abdominal computed tomography (CT) or magnetic resonance imaging (MRI) within 4 weeks before the anti-PD-1/PD-L1 immune therapy. 5) at least once efficacy evaluation after 3 cycles of anti-PD-1/PD-L1 treatment with enhanced MRI and CT. Exclusion criteria were: 1) less than 18 years old, 2) individuals with secondary liver cancer, cholangiocarcinoma, mixed liver cancer, or particular types of liver cancer, 3) combined with other tumors, 4) pregnant or lactating women, and 5) those without complete clinical laboratory data.

### 2.3 Human plasma collection and preparation

Fasting venous blood was collected. The plasma was collected by centrifuging blood samples and then stored at -80°C until UHPLC-MS analysis. All frozen plasma samples were thawed at 4°C. Afterwards, 600 μL of acetonitrile was added to 200 μL of plasma in ice. Mixtures were shaken at 3,000 rpm for 30 seconds to make them evenly mixed and then centrifuged at 13,000 rpm for 10 min at 4°C to remove protein. Then, supernatants were dried with nitrogen blow and stored at -80°C. Before analysis, each sample was reconstituted with acetonitrile/water (80:20, v/v) solution. Quality control (QC) samples were prepared by pooling 20 μL of each plasma sample.

### 2.4 Follow-up and therapeutic response assessment

All enrolled patients were followed up continuously, and the best of response (BOR) and survival data were recorded. Treatment response was assessed once every 3 cycles of anti-PD-1/PD-L1 treatment (around every 9-12 weeks) according to the Response Evaluation Criteria in Solid Tumors (RECIST V1.1), or whenever a sign or symptom suggested tumor progression. Anti-PD-1/PD-L1 therapy was continued until disease progression, intolerable adverse events, or discontinuation at the doctors’ discretion. Follow-up continued until the patient’s death or was lost to follow-up.

Complete response (CR) was defined as the disappearance of all target lesions, and the short diameter of all pathological lymph nodes had to be reduced to less than 10 mm. Partial response (PR) was defined as a reduction in the sum of the diameters of the target lesions of 30% or more (based on the baseline) for at least 4 weeks. Stable disease (SD) was defined as the degree of reduction of target lesion diameter that did not reach PR or the degree of increase that did not reach progressive disease (PD). PD was defined as a relative increase in the sum of diameters of target lesions of at least 20% (based on the minimum of the sum of diameters of all measured target lesions over the course of treatment), in addition to an absolute increase in the sum of diameters of at least 5 mm (the appearance of new lesions was also considered as progression). Disease control rate (DCR) refers to the percentage of cases with CR or PR or SD in the total number of evaluable cases in treatment. DCR (%) = (number of CR cases + number of PR cases + number of SD cases)/total number of evaluable cases x100%. Overall survival (OS) was the time from the start of therapy to death or loss to follow-up from any cause, and progression-free Survival (PFS) was the time from the start of therapy to the first tumor progression or death.

### 2.5 Analyst of plasma by UHPLC-MS

An UltiMate™ 3000 basic automated system (Thermo Scientific, Waltham, USA) coupled with an Orbitrap Fusion™ Tribrid™ mass spectrometer (Thermo Scientific, San Jose, CA, USA) was used for untargeted metabolomics analysis. The chromatography column used an Accucore™ HILIC HPLC column (100×2.1mm i.d., 2.6μm), the column temperature was set at 35°C, and the flow rate was 0.3 mL/min. The injection volume was 10 μL. Mobile phase A consisted of water/acetonitrile/formic acid (95:5:0.1, v/v/v) containing 10 mM ammonium formate, and mobile phase B consisted of acetonitrile. The starting composition was 5% A, which was maintained for 2 min before being increased to 20% at 2.0 min and 80% at 20.0 min for a 1.0 min wash, followed by returning to 5% A in 1 min and held until 8 min for a re-equilibration step. The spray voltage was set at 3.5 kV in positive ion modes. The full MS scan range was set at *m/z* 70-1050 Th with a resolution setting of 120,000. The ion transfer tube temperature and vaporizer temperature were set at 320°C and 275°C, respectively. The sheath gas and aux gas were set at 35 psi and 10 psi, respectively. Collision induced dissociation (CID) experiments were carried out for MS/MS analysis. During the CID experiments, precursor ions were isolated with a window width of 1.6 Th, and normalized collision energy (NCE) was set at 25-35%. The QC samples were inserted into the running sequence after every 6 samples to monitor the stability of data acquisition.

### 2.6 Chemical identification and data analysis

Raw data of each sample were obtained by Xcalibur 3.0 software. MSConvert was used to convert all data into mzML format data. Then, mzML data were uploaded to MetaboAnalyst 5.0 (https://www.metaboanalyst.ca/) and data processing was performed in the Spectra Processing module of LC-MS. The resulting peak intensities were used for statistical and functional analysis. Principal component analysis (PCA) and partial least-squares discriminant analysis (PLS-DA) analysis of plasma samples data obtained above were performed using MetaboAnalyst 5.0 software. The robustness, predictive capacity, and validity of the PLS-DA model were also confirmed using R (2), Q (2) parameters, prediction accuracy, and permutation tests. The differential metabolites that satisfied the criterion of variable importance in the projection (VIP) of >1.0 and p-value of <0.05 were considered as potential metabolic biomarkers. The biomarkers were putatively annotated based on mass measurement and their fragmentation patterns via CID as well as by consulting databases of the Human Metabolome Database (HMDB, http://www.hmdb.ca) and the LIPID MAPS (http://www.lipidmaps.org) to further enhance the accuracy of biomarker identification. Advanced heatmap plots, clustering correlation heatmap with signs, and correlation network were performed on the identified biomarkers using the OmicStudio tools at https://www.omstudio.cn to find the relationship and interaction mechanism between the markers.

## 3 Results

### 3.1 Patient baseline characteristics

Fifty-nine HCC patients who received anti-PD-1/PD-L1 therapy were enrolled for analysis. There were 44 men (74.58%) and 15 women (25.42%), with a median age of 58 years (52-66 years). The ECOG PS score of all patients was 0 (47.46%) or 1 (52.54%). Forty-four patients were classified as Child-Pugh A (74.58%), and 15 patients were classified as Child-Pugh B (25.42%). There were 42 patients (71.19%) with distant metastasis and 17 (28.81%) with local advanced. Nineteen patients (32.20%) received first-line treatment and 40 patients (67.80%) received non-first-line treatment. Regarding anti-PD-1/PD-L1 therapy, 55 patients (93.22%) received anti-PD-1/PD-L1 therapy combined with targeted therapy, and 4 (6.78%) received anti-PD-1/PD-L1 therapy combined with chemotherapy. There were 0 patients (0%) with complete response (CR), 20 patients (33.9%) with partial response (PR), 27 patients (45.8%) with stable disease (SD), and 12 patients (20.3%) with progressive disease (PD). The baseline characteristics are listed in **Table 1**.

**Table 1:** The baseline characteristics of the study population.

Variables	Total (n=59)	PR (n=20)	SD (n=27)	PD (n=12)	P
Ages, years, (median (IQR))	58 (52-66)	58 (50-66)	58 (55-66)	59 (48-65)	0.847
Sex, male/female, n (%)	44/15 (74.58/25.42)	13/7 (65.00/35.00)	21/6 (77.78%/22.22)	10/2 (83.33/16.67)	0.450
BMI, kg/m2, (median (IQR))	23.44 (20.79-25.83)	24.53 (20.25-25.90)	23.03 (20.52-24.80)	22.96 (20.97-26.12)	0.497
First-line treatment, yes/no, n (%)	19/4 (32.20/67.80)	7/13 (35.00/65)	8/19 (29.63/70.37)	4/8 (33.33/66.67)	0.923
AFP, (median (IQR))	243.30 (13.52-7548.00)	205.30 (13.52-9714.50)	243.3 (3.71-3576.2)	668.1 (23.86-60000)	0.471
Hb, g/L, (median (IQR))	131.00 (119.00-143.00)	138.00 (121.00-145.75)	130.00 (113.00-136.00)	147.00 (126.25-156.25)	0.035
Pltelet, ×109/L, (median (IQR))	169.00 (117.00-230.00)	175.00 (132.00-228.50)	166.00 (97.00-241.00)	151.50 (121.25-199.50)	0.855
INR, (median (IQR))	1.03 (0.97-1.10)	1.02 (0.97-1.06)	1.03 (0.97-1.12)	1.03 (0.95-1.10)	0.769
ALT, U/L, (median (IQR))	22.50 (16.30-37.30)	24.90 (15.3-39.40)	20.90 (16.30-31.90)	30.95 (20.75-41.80)	0.357
AST, U/L, (median (IQR))	29.60 (23.10-48.70)	29.85 (18.63-53.88)	27.80 (19.60-41.90)	34.40 (23.15-63.35)	0.662
Albumin, g/dL, (median (IQR))	37.30 (34.30-41.30)	39.00 (35.50-41.55)	36.50 (22.80-40.90)	36.40 (32.75-38.93)	0.333
Tbil, μmol/L, (median (IQR))	17.10 (10.30-24.20)	12.15 (9.70-22.85)	13.90 (10.70-22.70)	19.35 (17.63-50.75)	0.095
Cirrhosis, yes/no, n (%)	25/34 (42.37/57.63)	9/11 (45.00/55.00)	10/17 (37.04/62.96)	6/6 (50.00/50.00)	0.427
Extrahepatic metastasis, yes/no, n (%)	42/17 (71.19/28.81)	14/6 (70.00/30.00)	19/8 (70.37/29.63)	9/3 (75.00/25.00)	0.948
Number of tumors, solitary/multiple, n (%)	14/45 (23.73/76.27)	6/14 (30.00/70.00)	7/20 (25.93/74.07)	1/11 (8.33/91.67)	0.354
Largest tumor size, <5cm/≥5cm, n (%)	20/39 (33.90/66.10)	8/12 (40.00/60.00)	9/18 (33.33/66.67)	3/9 (25.00/75.00)	0.684
Portal vein thrombosis, yes/no	14/45 (23.73/76.27)	6/14 (30.00/70.00)	4/23 (14.81/85.19)	4/8 (33.33/66.67)	0.328
Child-Pugh grade, A/B, n (%)	44/15 (74.58/25.42)	17/3 (85.00/15.00)	21/6 (77.78/22.22)	6/6 (50.00/50.00)	0.078
ECOG PS, 0/1, n (%)	28/31 (47.46/52.54)	10/10 (50.00/50.00)	12/15 (44.44/55.56)	6/6 (50.00/50.00)	0.913

BMI, body mass index; AFP, alpha-fetoprotein; Hb, hemoglobin; INR, International Normalized Ratio; ALT, alanine aminotransferase; AST, aspartate aminotransferase; Tbil, total bilirubin.

### 3.2 Analysis of patient plasma samples by UHPLC-MS

**Figure 1:**
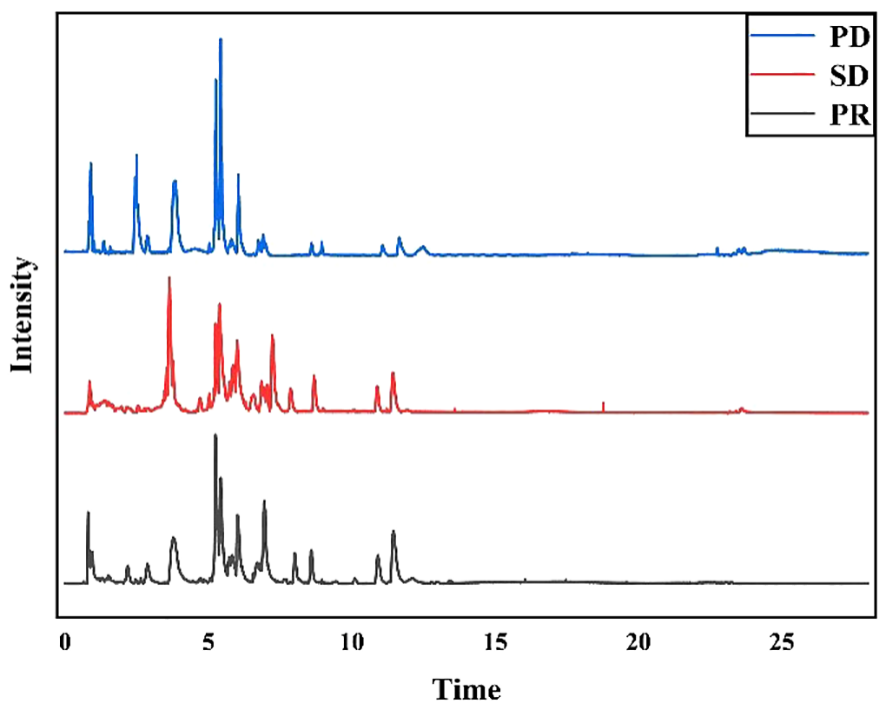
Typical total ion chromatograms of human plasma samples. PD group samples, SD group samples, and PR group samples in positive ion mode.

Fifty-nine plasma samples were analyzed by UHPLC-MS/MS in the positive ion mode. The typical total ion chromatograms of the PR, SD, and PD groups (**Figure 1**) show retention times in the range of 0 - 28 min. The quality of datasets was checked with PCA regarding gender (**Supplementary Figure S2A**) and age (**Supplementary Figure S2B**). Not surprisingly, no significant difference was found. To eliminate any artificial differences introduced by grouping patients, the HCC plasma sample data were scrambled, keeping each sample’s data intact and only changing the sample grouping for analysis (**Supplementary Figure S2C**). PCA determined QC and revealed no outliers (**Supplementary Figure S2D**), indicating that data acquired during this analysis were stable.

### 3.3 Analysis of plasma from HCC patients

**Figure 2:**
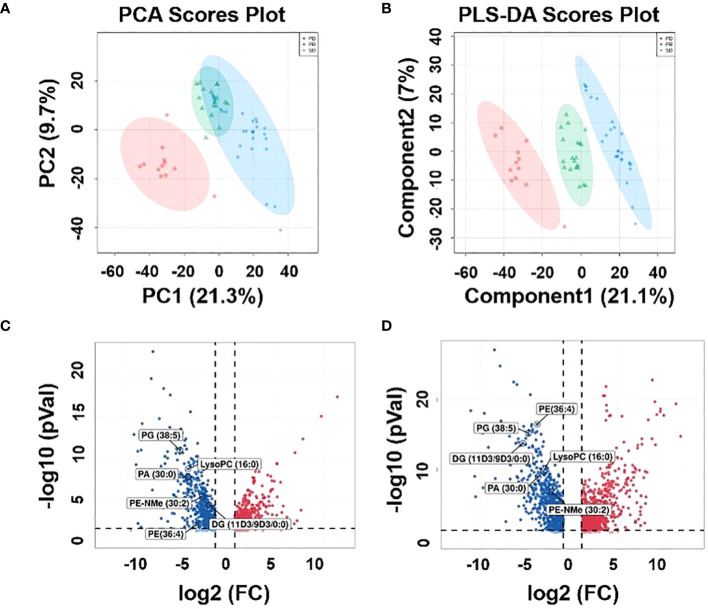
Metabolic profiles of the plasma samples. **(A)** Score plot of PCA, **(B)** score plot of PLS-DA, (green circles: PR, blue circles: SD, red circles: PD). **(C)** Volcano plot between PD plasma samples and PR plasma samples, **(D)** volcano plot be-tween PD plasma samples and SD plasma samples.

An untargeted metabolomics approach was first performed to build an unsupervised PCA model comprising plasma samples from 59 patients (including 27 SD plasma samples, 12 PD plasma samples, and 20 PR plasma samples). As revealed by the score plot of PCA (**Figure 2A**), the molecular patterns obtained by UHPLC-MS data from the three groups were clustered but did not show obvious separation trends between PD and SD. Thus, as an observation process, partial least squares-discriminant analysis (PLS-DA) can perform sorting and feature selectio (35). The PLS-DA score plot shows that three group specimens are not only completely separated from each other but clustered within the group (**Figure 2B**). To further validate the PLS-DA model, the best cross-validation tests (marked with red star) were performed, with intercepts R^2 =^ 0.993, Q^2 =^ 0.927, and prediction accuracy = 0.953 (**Supplementary Figure S3A**). Also, the permutation test was repeated 100 times, and the test was applied to get the p-value (p = 0.03) (**Supplementary Figure S3B**), indicating that the model was not overfitted. Moreover, variable importance in projection (VIP) values was calculated to evaluate the contribution of individual metabolites. Large VIP values > 1.0 were the most relevant for explaining differences between PD, SD, and PR groups. By comparing the MS/MS data of the HMBD and LIPID MAPS, 19 metabolites including [pyruvatoxime+H]^+^ (*m/z* 104.0165), [4-Ipomeanol+H]^+^ (*m/z* 169.0884), [amifostine+H]^+^ (*m/z* 215.0184), [DG(O-18:0/2:0/0:0)+NH_4_-H_2_O]^+^ (*m/z* 386.3406), [LysoPC(16:0)+H]^+^ (*m/z* 496.3422), [3b,16a,21b,22a)-12-Oleanene-3,16,21,23,28-pentol-22-angeloyloxy-23-al+NH_4_]^+^ (*m/z* 604.4204), [PA(30:0)+ NH_4_-H_2_O]^+^ (*m/z* 620.4405), [saponin H+NH_4_-H_2_O]^+^ (*m/z* 650.3941), [DG(11M3/9M5/0:0)+H]^+^ (*m/z* 673.5024), [PE-NMe(30:2)+H]^+^ (*m/z* 674.46), [DG(11D3/9D3/0:0) + NH_4_]^+^ (*m/z* 690.5242), [DG(42:10) +NH_4_]^+^ (*m/z* 706.5234), [PE(32:1) + NH_4_]^+^ (*m/z* 707.5269), [PA(37:4)+NH_4_-H_2_O]^+^ (*m/z* 710.4937), [PE(36:4)+H]^+^ (*m/z* 740.5445), [PG(36:4)+H-H_2_O]^+^ (*m/z* 753.5169), [PE(38:9)+NH_4_-H_2_O]^+^ (*m/z* 757.4687), [PG(38:5)+H]^+^ (*m/z* 797.5432), and [PG(40:6)+H]^+^) (*m/z* 840.5657) identified in plasma samples had VIP>1.5, and 14 metabolites had VIP values >2 (**Supplementary Figure S4**; **Supplementary Table S1**). Volcano plots can perform the distribution of metabolites with differential expression based on P-value (p < 0.05) and fold change (FC > 2) (36). Therefore, the results of the volcano plots were compared for the analysis of the PD group with the PR and SD groups, respectively. The volcano plots show that the lipid metabolites with higher VIP values are significantly distributed (**Figures 2C, D**). These results suggested that the corresponding metabolites might act as potential biomarkers predicting the therapeutic effect of HCC patients.

### 3.4 Correlation cluster analysis and metabolic analysis visualization of nineteen metabolites in HCC plasma samples

**Figure 3:**
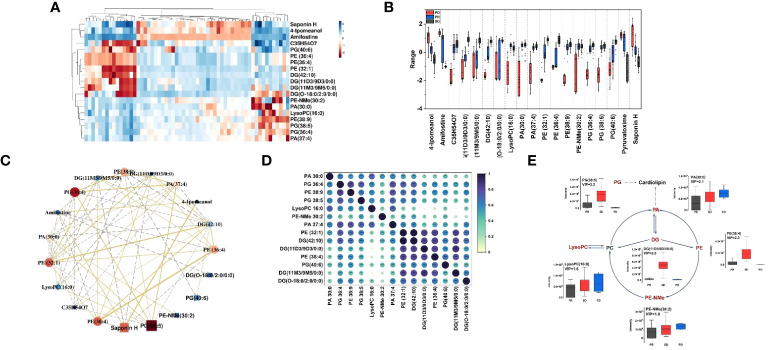
**(A)** Heatmap constructed based on the UHPLC-MS signal intensities of identified 19 metabolites, with a high VIP value in differential analysis of HCC plasma samples. Dark red represents relative high abundances of biomarkers in the sample, and dark blue represents relative low abundances; **(B)** Boxplots for the relative abundances of 19 features among the different HCC groups in positive mode. Boxplots of the differential metabolites for the PD group (red), PR (blue) and SD (black); box plots display median (line within box) and quartiles (box limits); **(C)** Correlation network. The golden line represents a positive correlation between biomarkers, the gray line represents a negative correlation. The darker the red of the biomarker, the stronger the correlation in the network diagram, and the darker the dark blue, the weaker the correlation in the network diagram. The size of each ball can also reflect this. **(D)** Correlation coefficient heat map of 14 metabolites in the glycerophospholipid pathway. **(E)** The changed lipid metabolites were mapped to glycerophospholipid pathways according to their annotation in the KEGG database. The six lipid metabolites boxplots were changed obviously in the three groups of metabolic pathway.

To further compare the alterations of major iconic signals in the human model of immune therapy of HCC, the UHPLC-MS signal intensities of the 19 iconic signals which made the major contribution to the differentiation between different types of plasma according to the VIP values were extracted for the construction of heatmaps (**Figure 3A**). The heatmap reveal the signal intensity of metabolites in three groups, and each small cell represents the signal intensity of a single metabolite in one sample. PE-NMe (30:2), PA (30:0), LysoPC (16:0), PE (36:4), PG (38:5), and DG (11D3/9D3/0:0) changed most obviously in the three groups of plasma samples, which had low P- value and high fold change (**Supplementary Figure S5**). Hence, investigated the metabolite alteration trends based on UHPLC-MS for different immunotherapeutic effects. Interestingly, the intensity of mass spectrometric signal responses of 19 metabolites correlated with the immunotherapeutic effect of HCC (**Figure 3B**). To explore the relationship between 19 metabolites, biomarker correlation analysis was performed showing significant correlations between metabolites (**Figure 3C**). The correlation network plots show more clearly relationship between each metabolite, where 14 lipid metabolites show a strong positive correlation with each other, with the significant number of correlated objects for PE-NMe (30:2), PA (30:0), LysoPC (16:0), PE (36:4), PG (38:5) and DG (11D3/9D3/0:0).

As shown in **Figure 3D**, spearman rank-order correlation coefficient is shown in the matrix. The test was used to determine if the correlation was significant (* P-value <= 0.05, ** P-value <= 0.01, *** P-value <= 0.001). 14 lipid metabolites were positively correlated, and the metabolites of the same type had the strongest correlation, followed by the metabolites that were directly affected by each other in the metabolic pathway. Moreover, 14 lipid metabolites were on the glycerophospholipid metabolic pathway. Thus, as shown in the metabolic pathway (**Figure 3E**), the black text indicates that no metabolites were detected, and the red text indicates that metabolites were significantly enriched. The box plots show the intensity changes of representative related lipid metabolites on the pathway which change most obviously in three groups of plasma samples, including PE-NMe (30:2), 620 PA (30:0), LysoPC (16:0), PE (38:9), PG (38:5) and DG (O-18:0/2:0/0:0) and trends for these six lipid biomarkers are evident in each group. Among them, LysoPC(16:0), PA (30:0), PE-NMe (30:2) show the same trend of intensity change with high intensity of pre-treatment metabolites and poor treatment effect in HCC patients and DG (O-18:0/2:0/0:0), PE (38:9), PG (38:5) show the same trend of intensity change, with low intensity of pre-treatment metabolites in HCC patients and better treatment effect. Moreover, decreased palmitic acyl (C16:0)–containing glycerophospholipids were positively associated with metastatic abilities of HCC cells (37). It was reported that the level of LysoPC(16:0) gradually decreased with the progression of HCC (38), which was consistent with results, the signal level of LysoPC (16:0) showed a gradual increase as the actual immune therapy combined with chemotherapy efficacy of the patients improved.

### 3.5 Clinical value of the fourteen lipid metabolites

**Figure 4:**
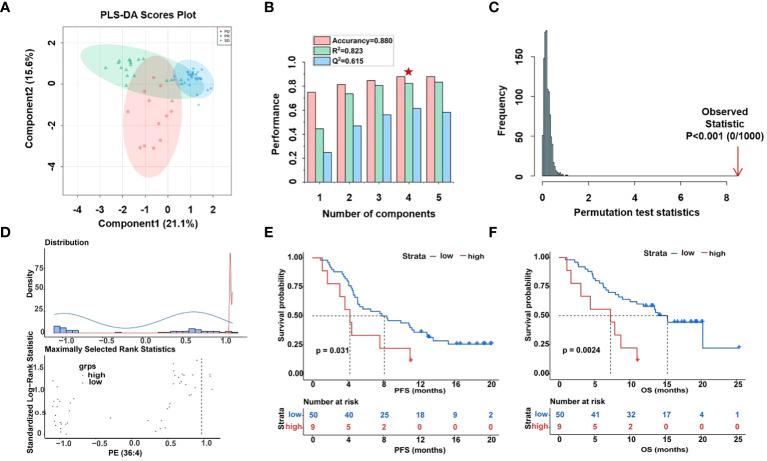
Differential analysis of fourteen lipid metabolites in plasma samples of HCC patients. **(A)** Score plot of PLS-DA models derived from UHPLC-MS data of PR (green circles), SD (blue circles), and PD (red circles), **(B)** cross-validation test (marked with a red star are the best explained and predicted parameteters) and **(C)** permutation test results (1000 permutations) of the PLS-DA model indicated that the model was not over-fitted. **(D)** Determining cut-off values and the plot of standardized log-rank statistics of PE (36:4), **(E, F)** the Kaplan-Meier plot according to the cut-off values of PE (36:4).

Over the last decade, immune checkpoint inhibitors (ICIs), such as PD-1, PD-L1, and others have dramatically changed the treatment algorithm for solid tumors. However, the role of existing predictive biomarkers, such as the expression of PD-L1, in predicting the prognosis of HCC receiving ICIs therapy remains to be clarified (39). Here, the potential value of 14 phospholipid metabolites (including DG (O-18:0/2:0/0:0), LysoPC (16:0), PA (30:0), DG (11M3/9M5/0:0), PE-NMe (30:2), DG (11D3/9D3/0:0), DG (42:10), PE (32:1), PA (37:4), PE (36:4), PG (36:4), PE (38:9), PG (38:5) and PG (40:6)) to predict the prognostic of HCC patients was again analyzed by PLS-DA (**Figure 4A**). Surprisingly, similar performance was obtained when using only the fourteen glycerophospholipid metabolites, the best explained parameters and predictive parameter (marked red star) of three groups PLS-DA models were obtained by cross-validation tests (**Figure 4B**), with intercepts R^2^ = 0.823, Q^2^ = 0.615, and prediction accuracy = 0.880. The permutation test was repeated 1000 times (**Figure 4C**), and the test was applied to get the p-value (p < 0.001), indicating that the model was not over-fitted. The obtained performance using the fourteen types of lipid metabolites was similar to the reported performance of the cross-validation tests concerning all metabolites (R^2^ = 0.998 vs. R^2^ = 0.823) but superior concerning the permutation test (p=0.03 vs. p < 0.001).

Moreover, optimal cut-off values for 14 glycerophospholipid metabolites based on standardized log-rank statistics. Patients were divided into the high and low metabolite groups according to the optimal cut-off of the relative abundance of each differential glycerophospholipid metabolites respectively. The Kaplan-Meier method was applied to analyze patients’ PFS and OS in the high and low metabolite groups. And the results of the Kaplan-Meier Survival analysis suggested that survival benefits were different between the high with low metabolite groups (**Figures 4D–F**, **Supplementary Figure S6**). In the Kaplan-Meier survival curve, the abscissa represents time, and the ordinate represents the progression-free survival rate or OS rate of patients. As shown in **Figures 4E, F**, both PFS and OS were significantly lower in the high metabolite group than in the low metabolite group. This indicated that metabolite PE (36:4) was closely related to the progression and mortality of HCC patients. Kaplan-Meier survival analysis were performed separately for 14 differential glycerophospholipid metabolites, and the results were similar to PE (36:4). The results showed that the survival curves of PFS and OS differed between high and low metabolite groups.

## 4 Discussion

The purpose of this study is to predict the therapeutic effect of HCC patients receiving anti-PD-1/PD-L1 therapy by an untargeted metabolomics based on UHPLC-MS. We proposed a method based on building a two-step PLS-DA prediction model strategy for finding the significative metabolites that can predict the efficacy of immune therapy for HCC in the following steps: in the first step, all metabolites in plasma samples are retained and biomarkers with characteristic properties are screened based on VIP values and a total of 19 metabolites were screened. In the second step, a prediction model was built from the screened biomarkers with prediction accuracy of 0.880. Moreover,14 biomarkers were identified, all which inside the glycerophospholipid pathway in HCC plasma samples. These biomarkers showed a high correlation with each other, which may make an essential contribution to the prediction of immune efficacy.

Lipids play a crucial role in regulating normal cellular function. Disorders of lipid metabolism have been associated with the development of various human diseases (40–42). Glycerophospholipids, as a type of lipid, play an important metabolic role in plasma (43). They are major components of cell membranes and are involved in maintaining cell structure and function. In this study, 14 biomarkers were related to glycerophospholipid metabolism, six biomarkers showed the greatest change in the significance of the signal (including PE-NMe (30:2), PA (30:0), LysoPC (16:0), PE (36:4), PG (38:5), and DG (11D3/9D3/0:0)) in PR patients and SD patients compared to PD patients. The therapeutic effect of anti-PD-1/PD-L1 on patients decreases with the increase of LysoPC, PA, and PE-NMe signals. However, PE, PG, and DG have a poor effect after treatment with low signals, and with the enhancement of signals, the treatment has some effect. To explore the possibility of clinical application of biomarkers, the Kaplan-Meier Survival analysis showed two biomarkers that should receive more attention, PE (36:4) and DG (11M3/9M5/0:0), while PE (36:4) was one of the biomarkers with the most significant changes in signal intensity. Furthermore, metabolomics research has demonstrated that irregularities in glycerophospholipid metabolism can result in liver damage (44).

Previous studies have shown that proteomics, MRI radiomics, and genomics on predicting anti-PD-1/PD-L1 therapy markers has made several advances (45–47). In addition, Zhang et al. found that plasma mevalonate levels were positively correlated with the therapeutic effect of anti-PD -(L)1 antibodies, and confirmed that plasma mevalonate could enhance tumor immunity (48). Although the metabolomic correlation in predicting the efficacy of immune therapy for HCC remains quite preliminary, it may potentially serve as a method to predict the efficacy of immune therapy for HCC. In this study, 14 metabolites were found to be associated with immunotherapy efficacy, overall survival and disease-free survival. Thus, we proposed that the up-regulation of those two kinds of metabolites (DG (11M3/9M5/0:0), and PE (36:4)) patients with worse prognosis may be related to the enhanced metabolism of tumor cells in this population. These metabolites may be produced or secreted by tumor cells with an active metabolism, or they may be part of the tumor cell structure and released into the plasma of patients (39). In addition, those metabolites (including DG (O-18:0/2:0/0:0), LysoPC (16:0), PA (30:0), PE-NMe (30:2), DG (11D3/9D3/0:0), DG (42:10), PE (32:1), PA (37:4), PG (36:4), PE (38:9), PG (38:5) and PG (40:6)) were down-regulated in patients with worse prognosis, which may be due to mutations in tumor suppressor genes, resulting in down-regulation of its expression products, or due to suppression of normal hepatocyte metabolism (49–52). Thus, we should pay more attention to patients with a high relative abundance of those two kinds of metabolites (DG (11M3/9M5/0:0) and PE (36:4)), and low relative abundance of other metabolites, and imaging changes should be closely monitored to identify tumor progression in early stage. Timely adjustment of treatment or symptomatic treatment should be given to patients to improve the survival prognosis and quality of life.

In conclusion, this study demonstrated a two-step PLS-DA model strategy based on the application of untargeted metabolomics (UHPLC-MS), which can afforded new and valuable metabolites regarding to predict the therapeutic outcome of HCC patients receiving of this disease. This study identified metabolites strongly associated with the efficacy of HCC anti-PD-1/PD-L1 immune therapy (DG (11M3/9M5/0:0, PE (36:4), DG (O-18:0/2:0/0:0), LysoPC (16:0), PA (30:0), PE-NMe (30:2), DG (11D3/9D3/0:0), DG (42:10), PE (32:1), PA (37:4), PG (36:4), PE (38:9), PG (38:5) and PG (40:6)). The results could be utilized as a reference for further clinical examination. In response to the growing demand on predicting immune therapy in HCC, a more comprehensive analysis of HCC could be achieved at both protein and gene levels when combining with the determination of some other biomarkers in the future. Other metabolite profiling techniques, using either targeted or untargeted metabolomics in diverse sample matrices, are necessary to replicate our findings and to provide greater insight into the metabolites associated with predicting immunological treatment of HCC.

## Data availability statement

The original contributions presented in the study are included in the article/**Supplementary Material**. Further inquiries can be directed to the corresponding authors.

## Ethics statement

The studies involving humans were approved by the first hospital of Jilin University. The studies were conducted in accordance with the local legislation and institutional requirements. The participants provided their written informed consent to participate in this study.

## Author contributions

BL: Conceptualization, Data curation, Formal analysis, Investigation, Methodology, Project administration, Resources, Validation, Visualization, Writing – original draft, Writing – review & editing. JS: Conceptualization, Data curation, Formal analysis, Investigation, Methodology, Project administration, Resources, Validation, Visualization, Writing – original draft, Writing – review & editing. RS: Conceptualization, Writing – original draft, Writing – review & editing. RZ: Conceptualization, Writing – original draft, Writing – review & editing. FX: Conceptualization, Writing – original draft, Writing – review & editing. YZ: Conceptualization, Supervision, Writing – original draft, Writing – review & editing. NW: Conceptualization, Project administration, Supervision, Writing – original draft, Writing – review & editing. HC: Conceptualization, Writing – original draft, Writing – review & editing. SF: Conceptualization, Writing – original draft, Writing – review & editing.
