# Supplementary material for: Predicting effect of anti-PD-1/PD-L1 inhibitors therapy for hepatocellular carcinoma by detecting plasma metabolite based on UHPLC-MS
Source: Front Immunol. 2024 Apr 18;15:1370771. doi: 10.3389/fimmu.2024.1370771 (PMC11067499; doi:10.3389/fimmu.2024.1370771)
Supplement: Supplementary file 1 [file DataSheet_1.docx]

**
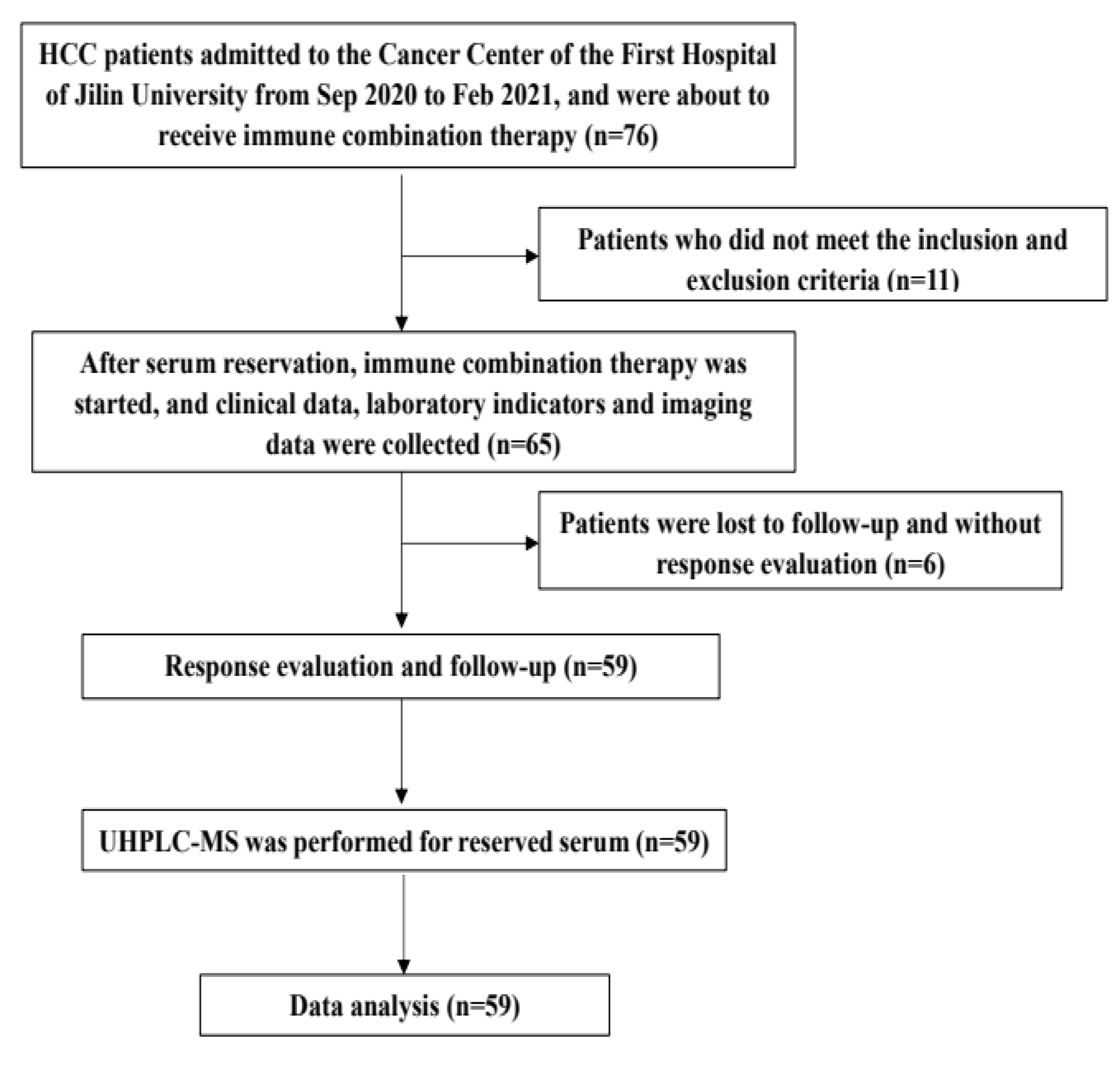
**

Figure S1 Workflow diagram of patient screening.


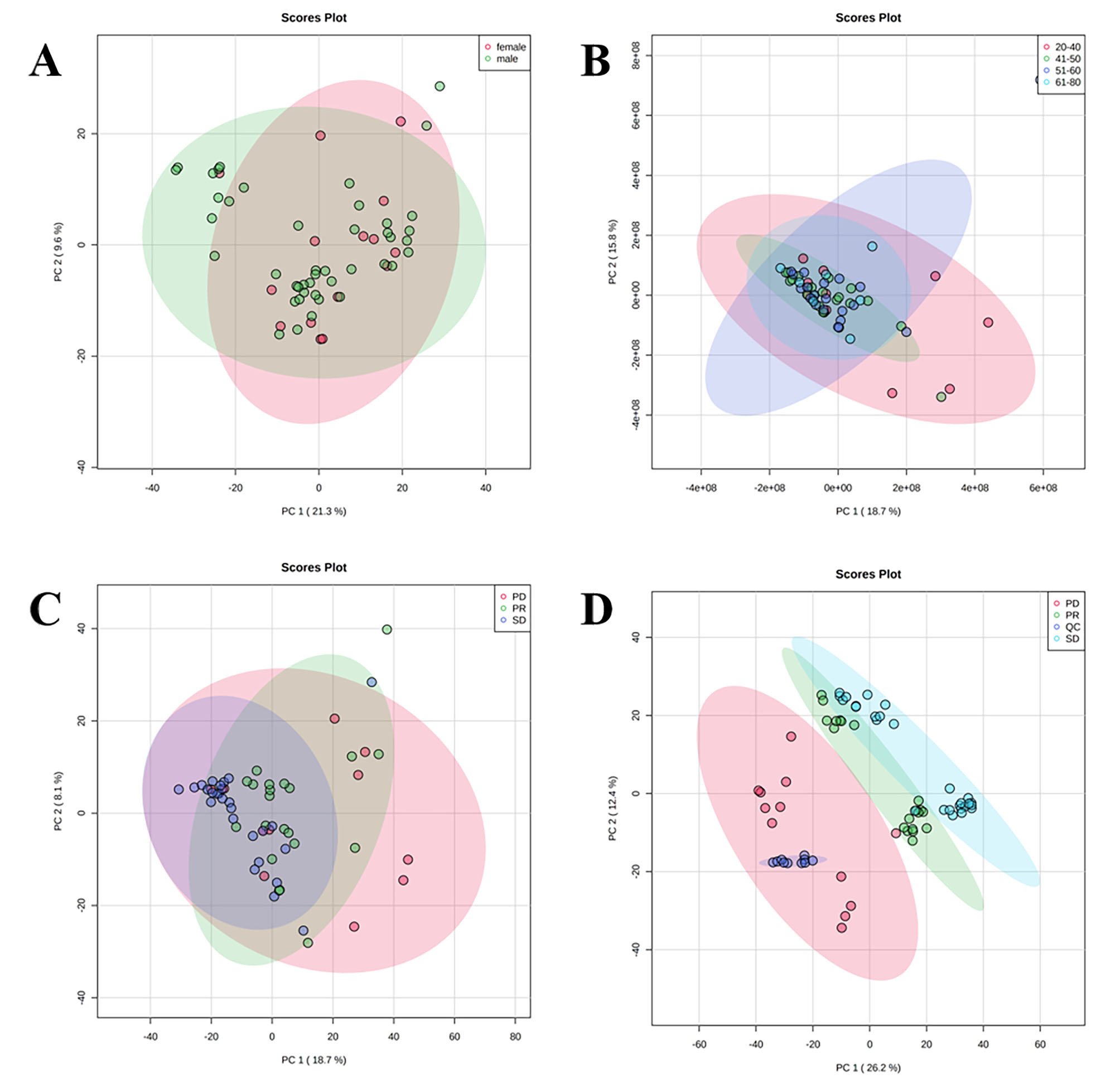


Figure S2 PCA excluded factors that may have influenced the experimental results: (A) gender; (B) age group; (C) disordered scramble rearrangement. PCA verifies data quality. (D) Quality control.


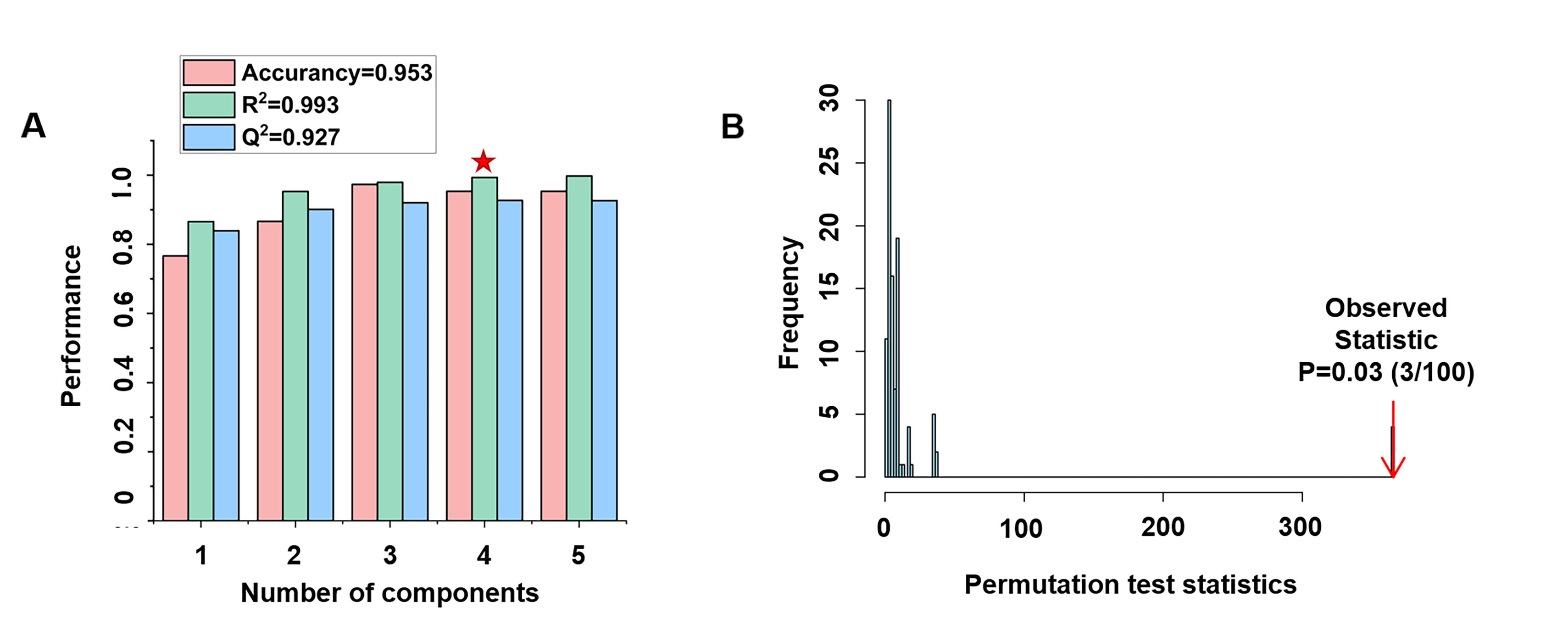


Figure S3 Differential analysis of plasma samples of HCC cancer patients. (A) cross-validation test and (B) permutation test results (100 permutations) of the PLS-DA model indicated that the model was not over-fitted


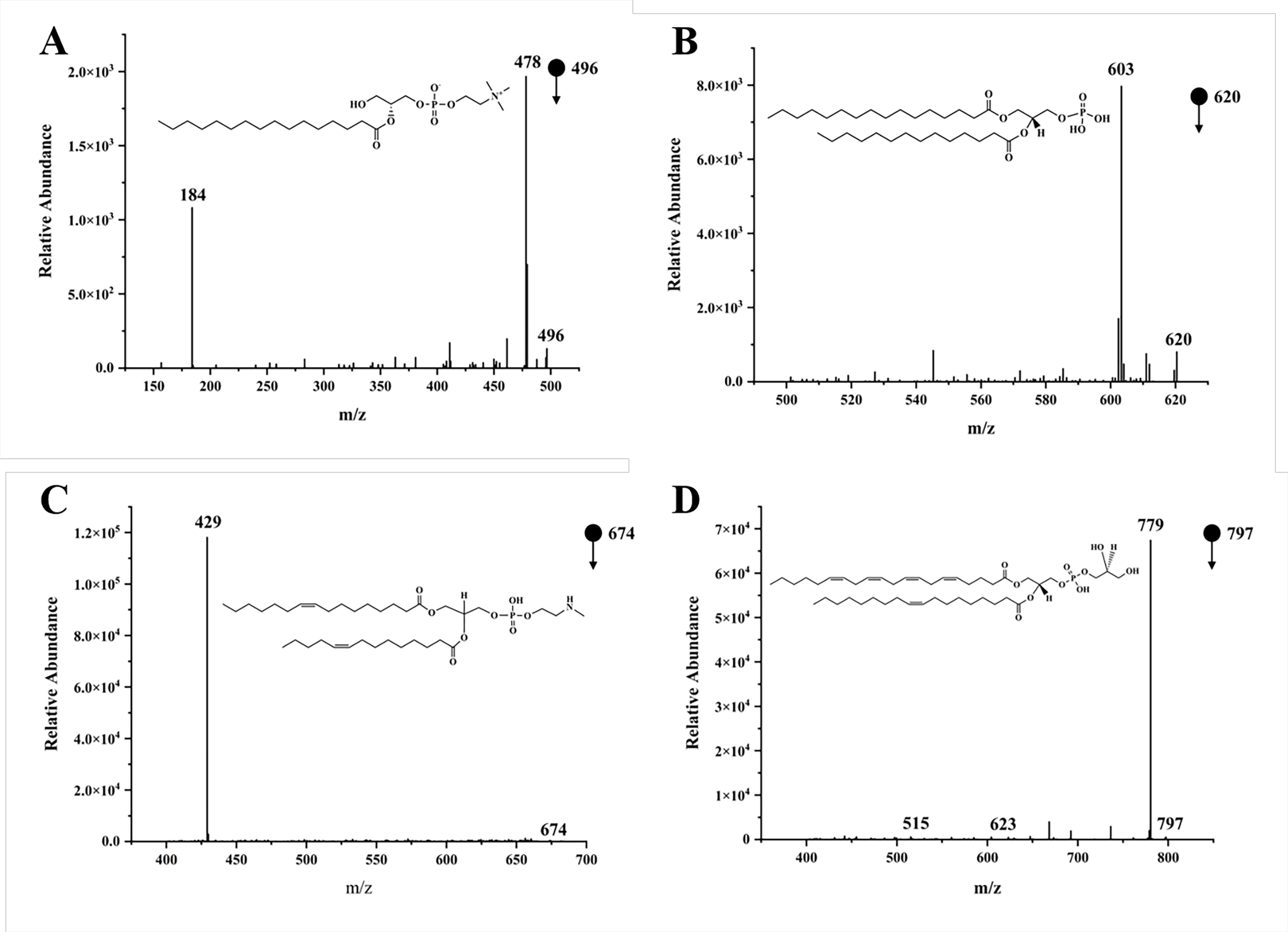


Figure S4 UHPLC-MS/MS spectra of (A) LysoPC(16:0), (B) PA(30:0), (C) PE-NMe(30:2), (D) PG(38:5) in plasma samples.


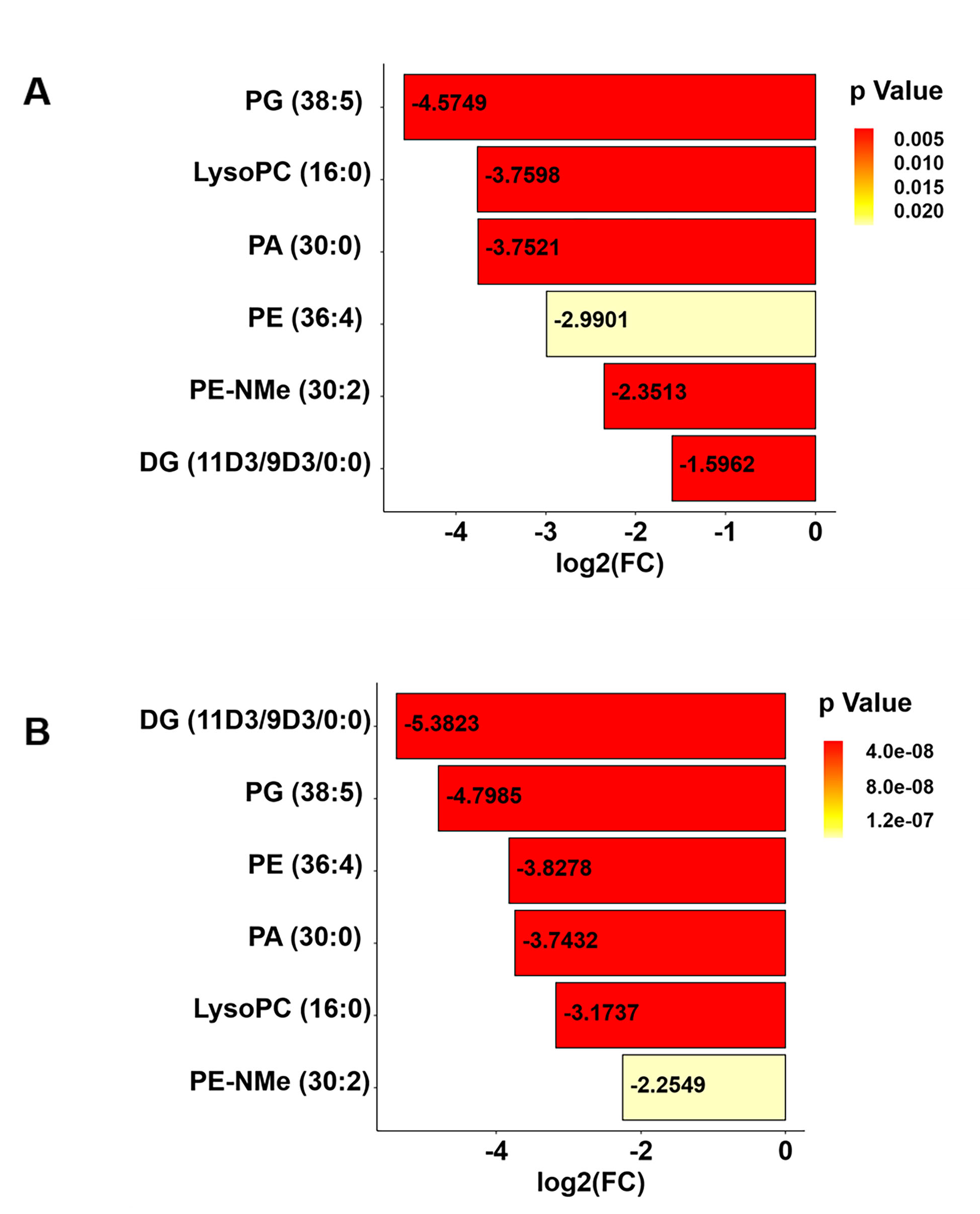


Figure S5 Metabolites of PE-NMe (30:2), PA (30:0), LysoPC (16:0), PE (36:4), PG (38:5) and DG (11D3/9D3/0:0) significance thresholds (FC > 2, p < 0.005), (A) PD and PR groups, (B) PD and SD groups.


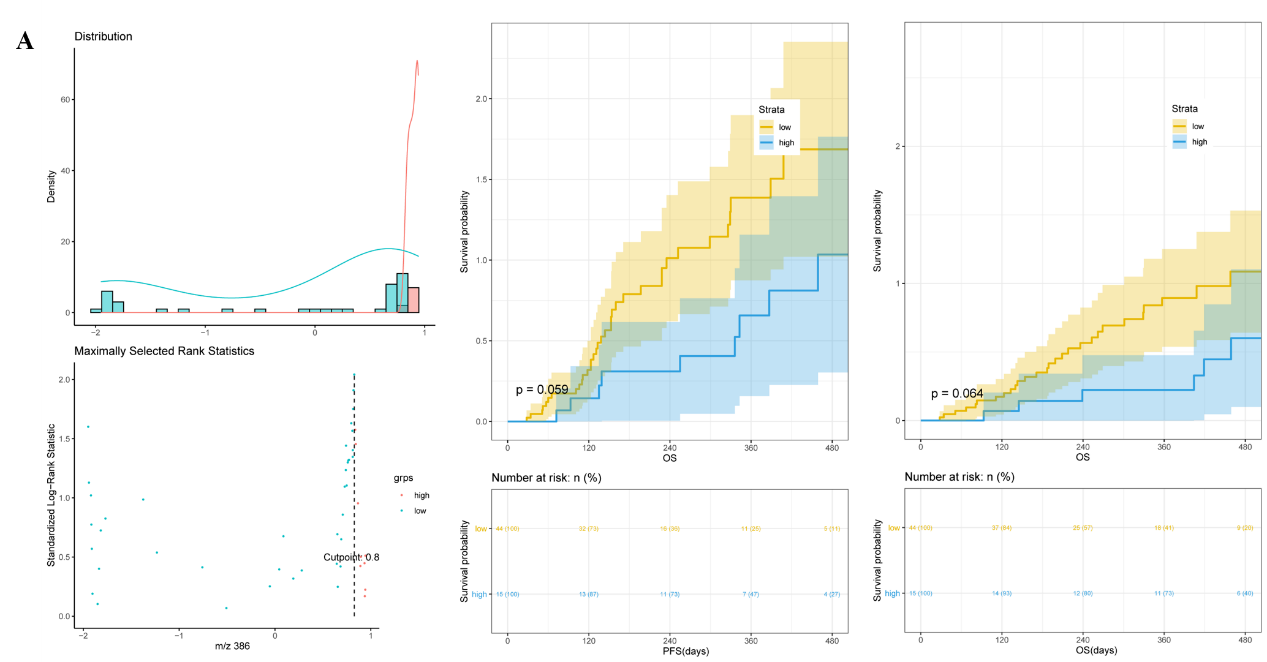

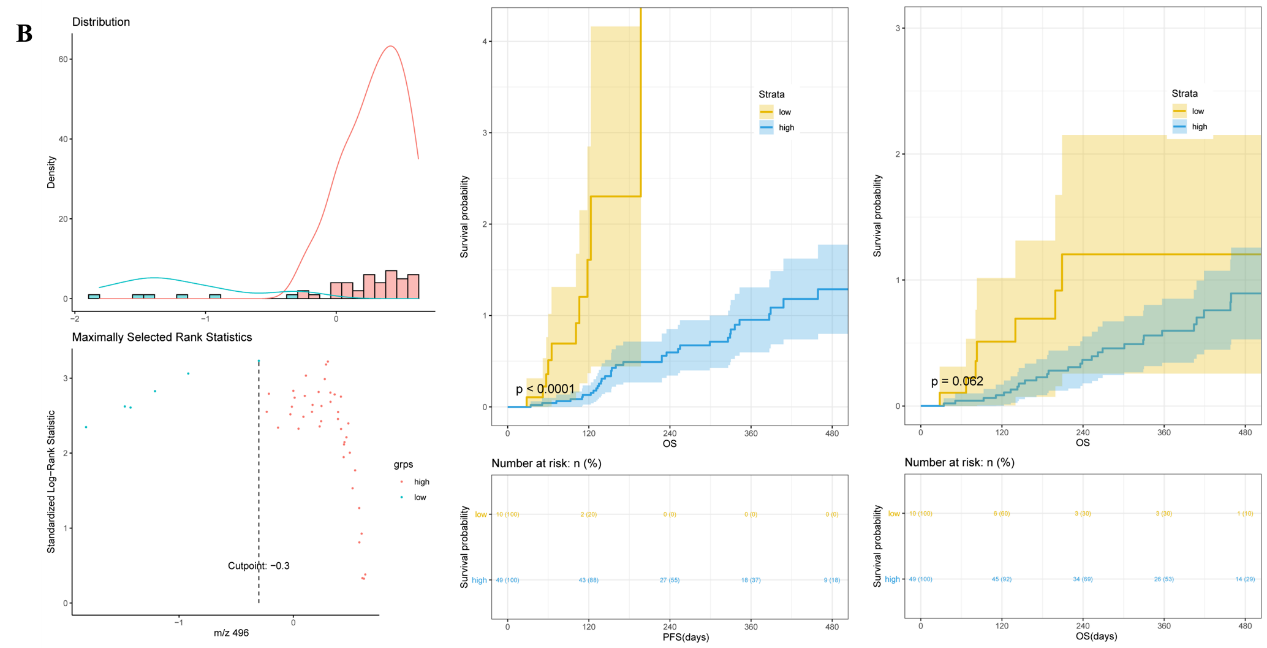

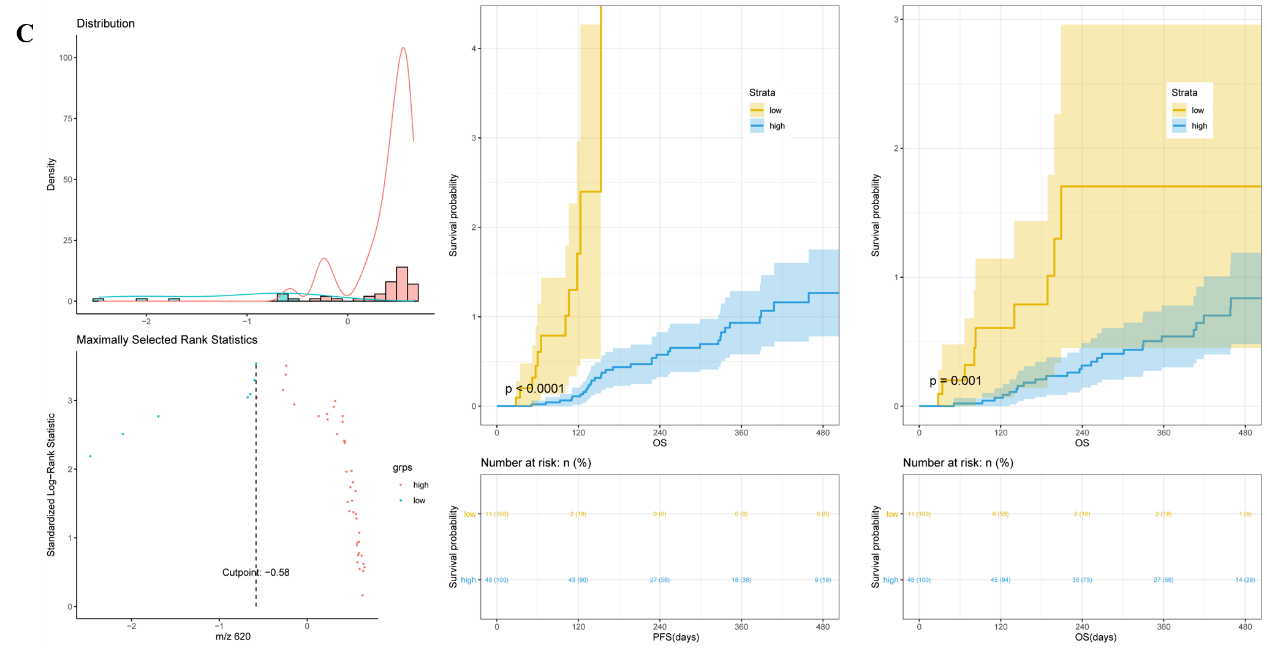

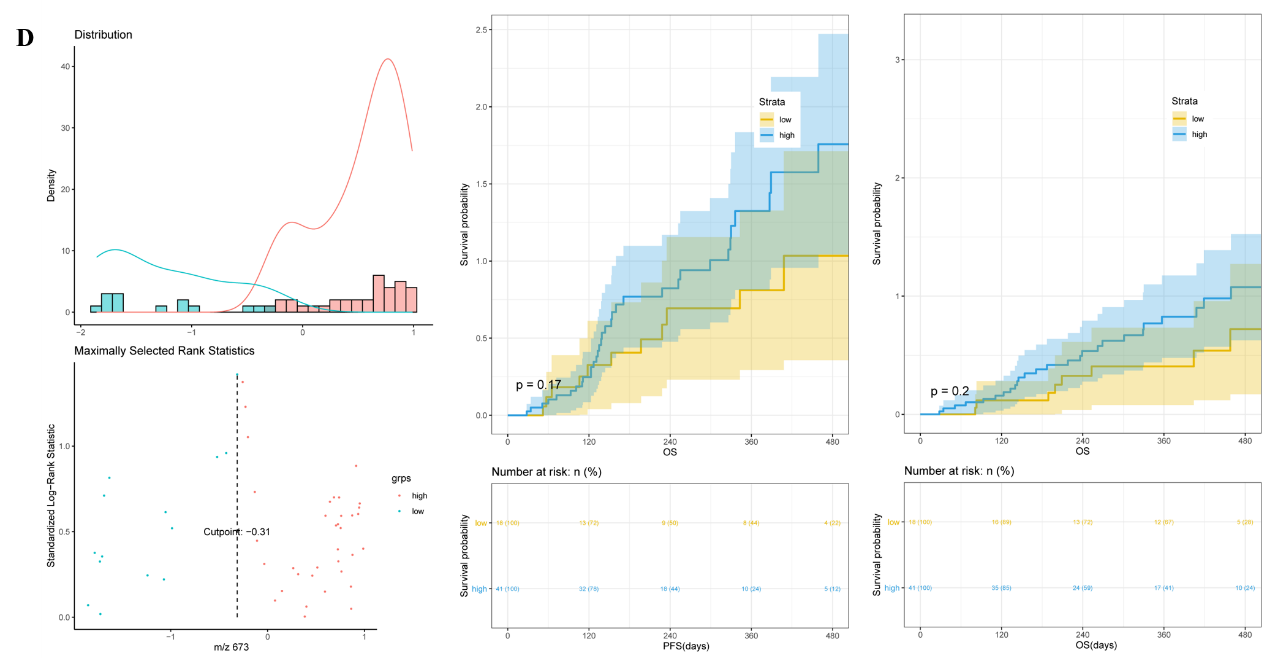

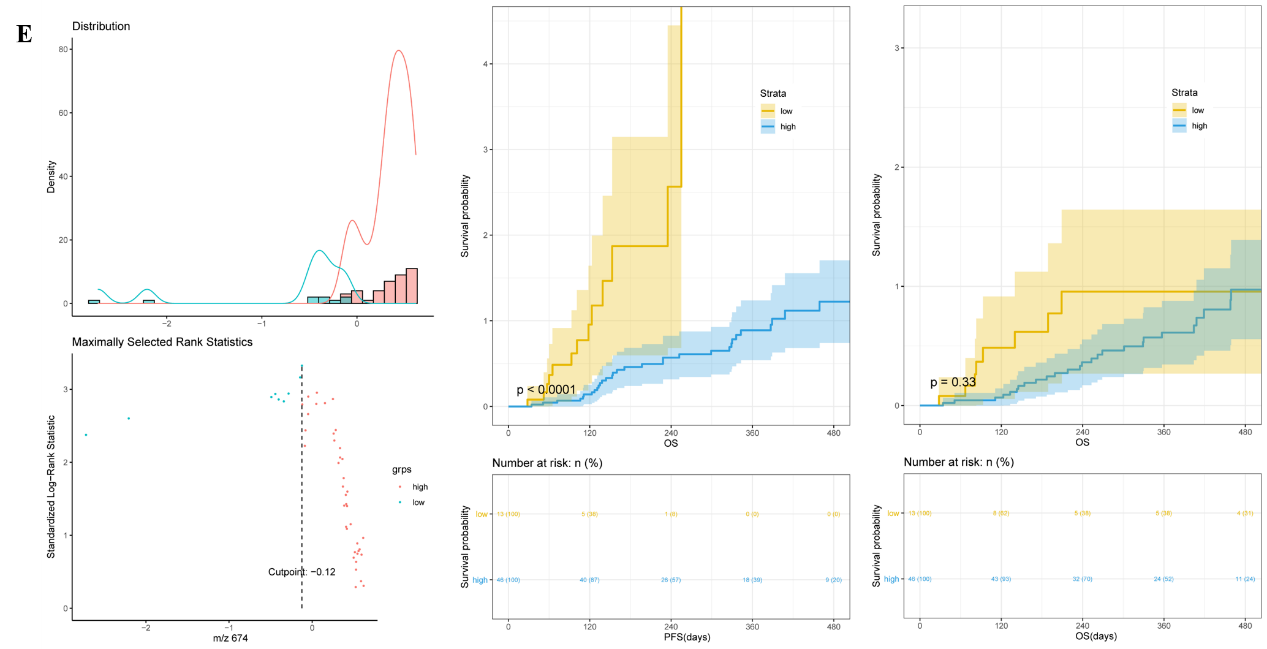

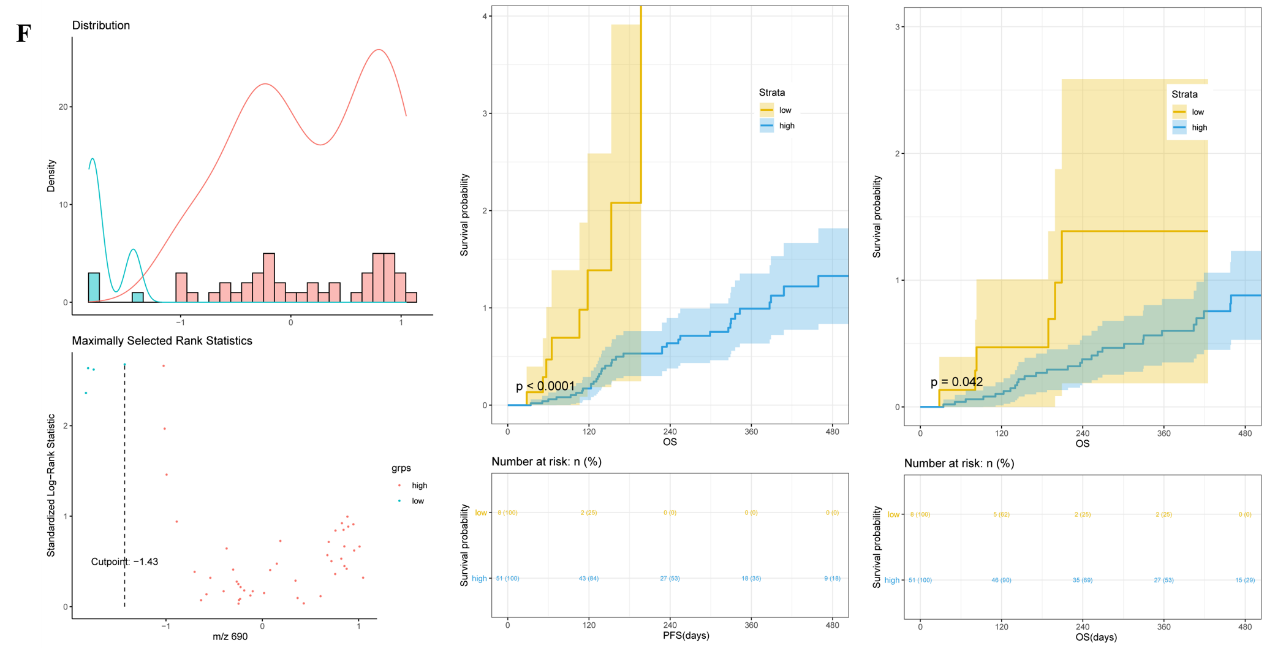

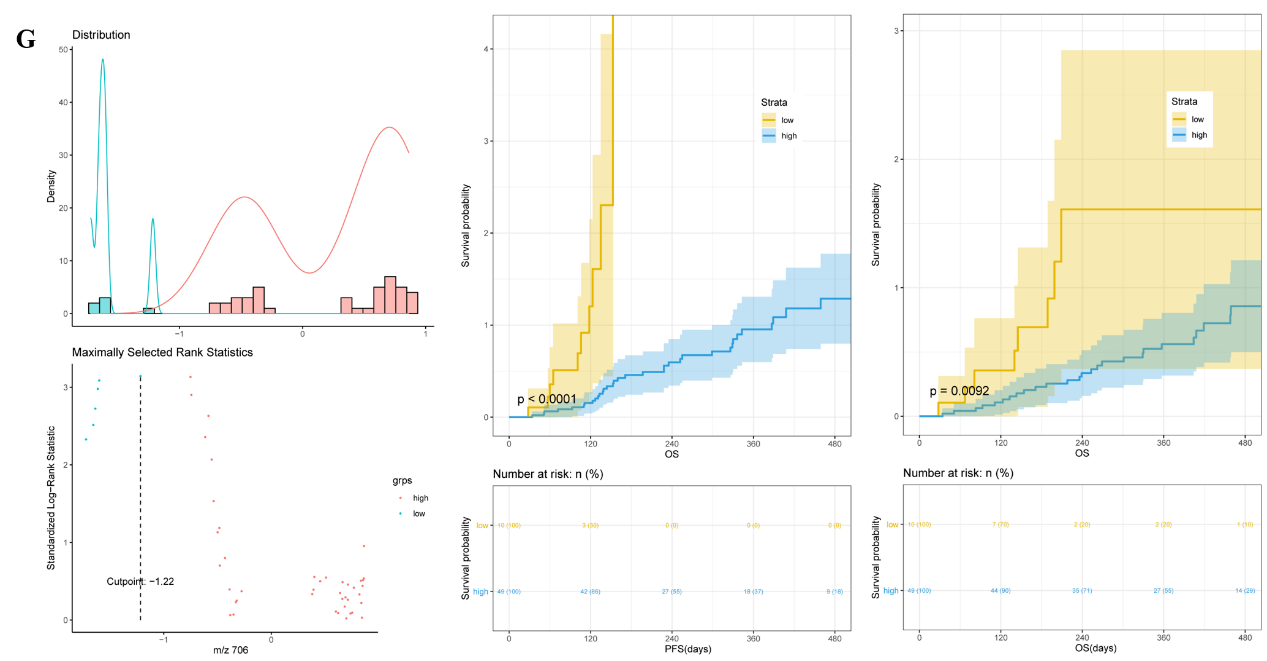

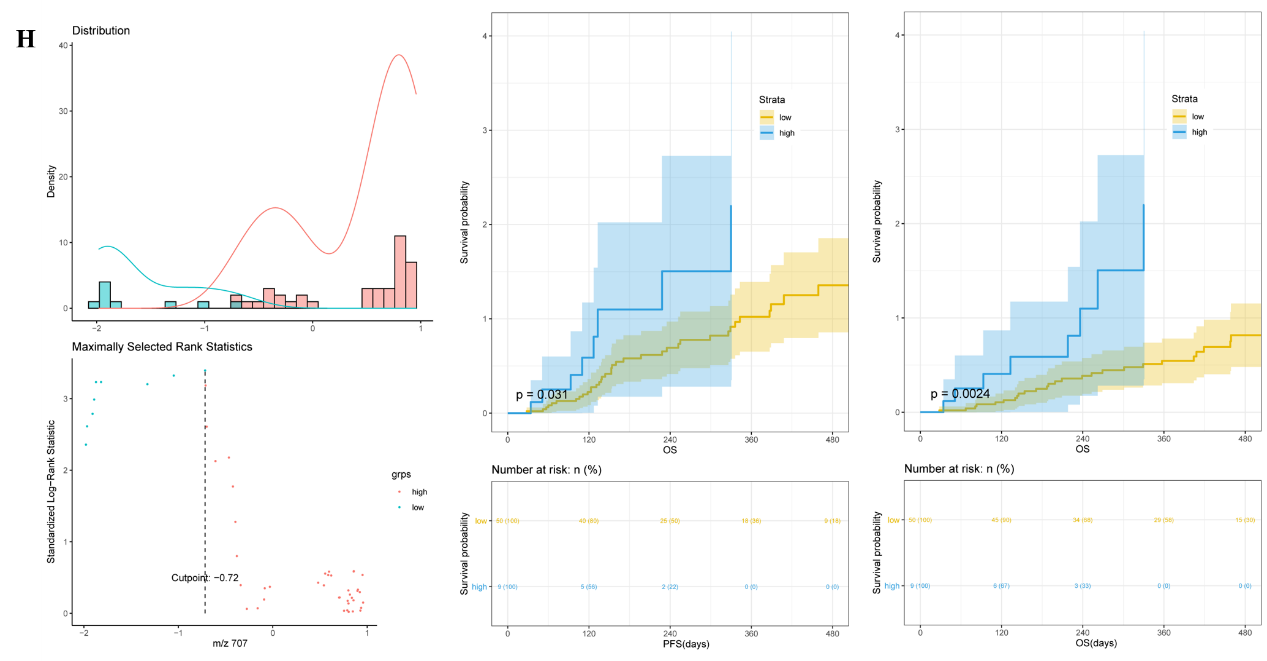

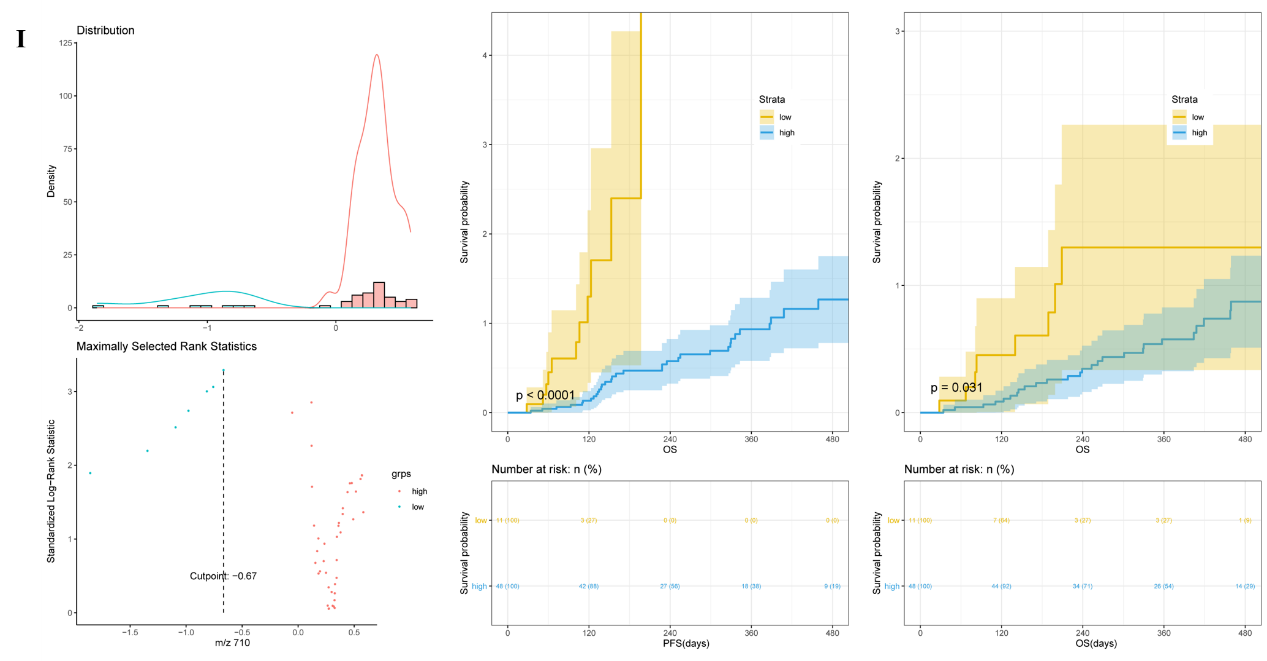

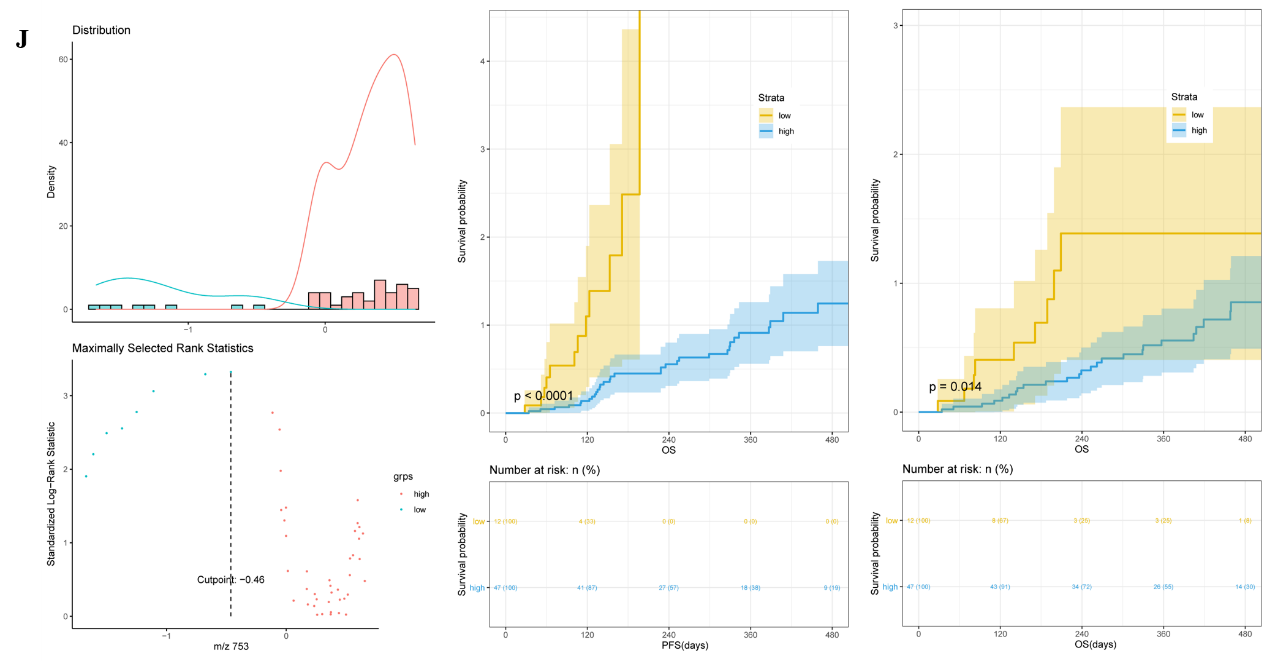

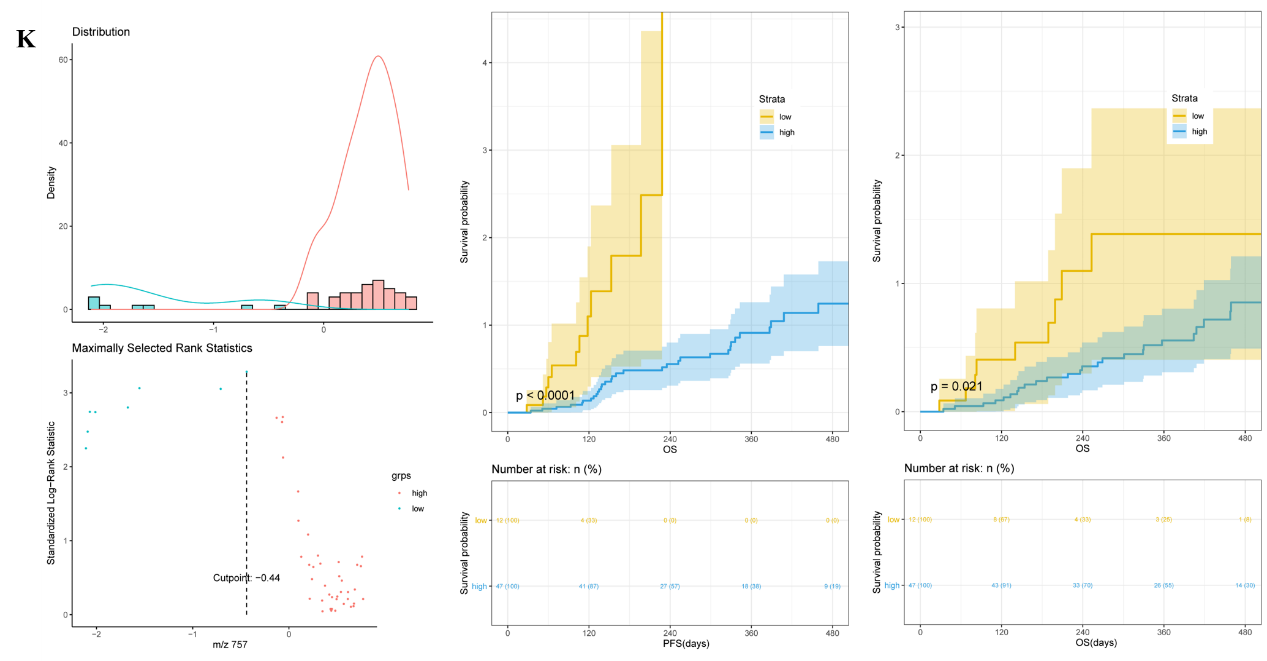

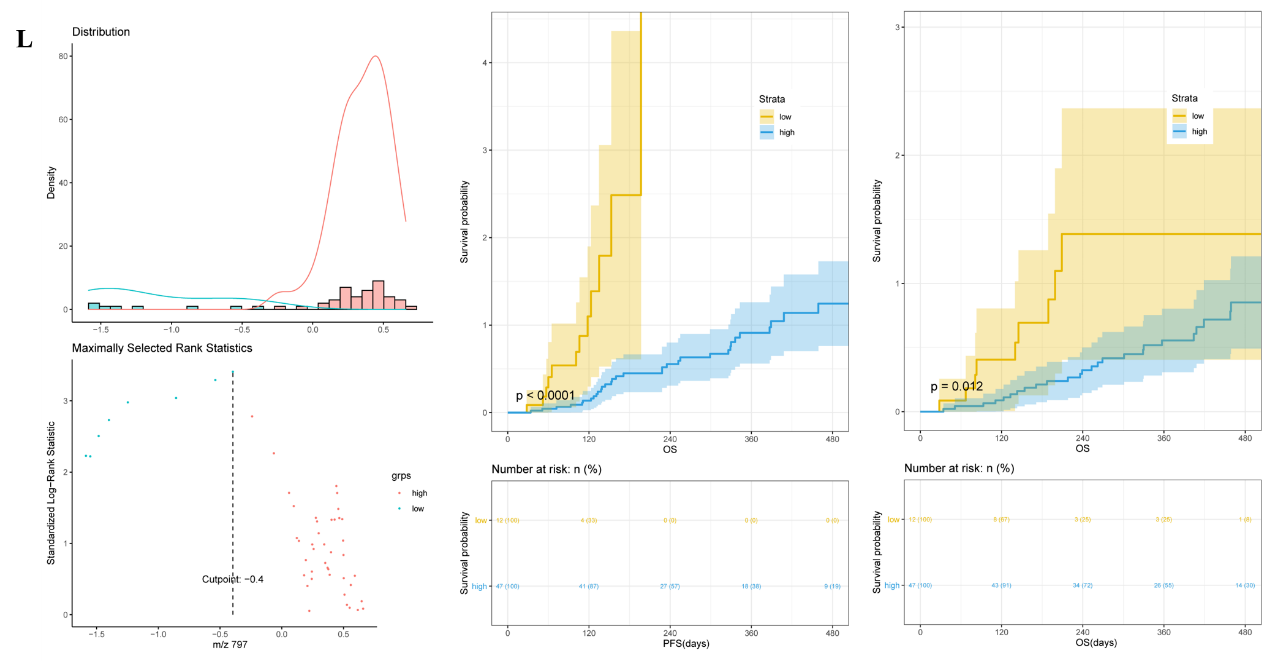

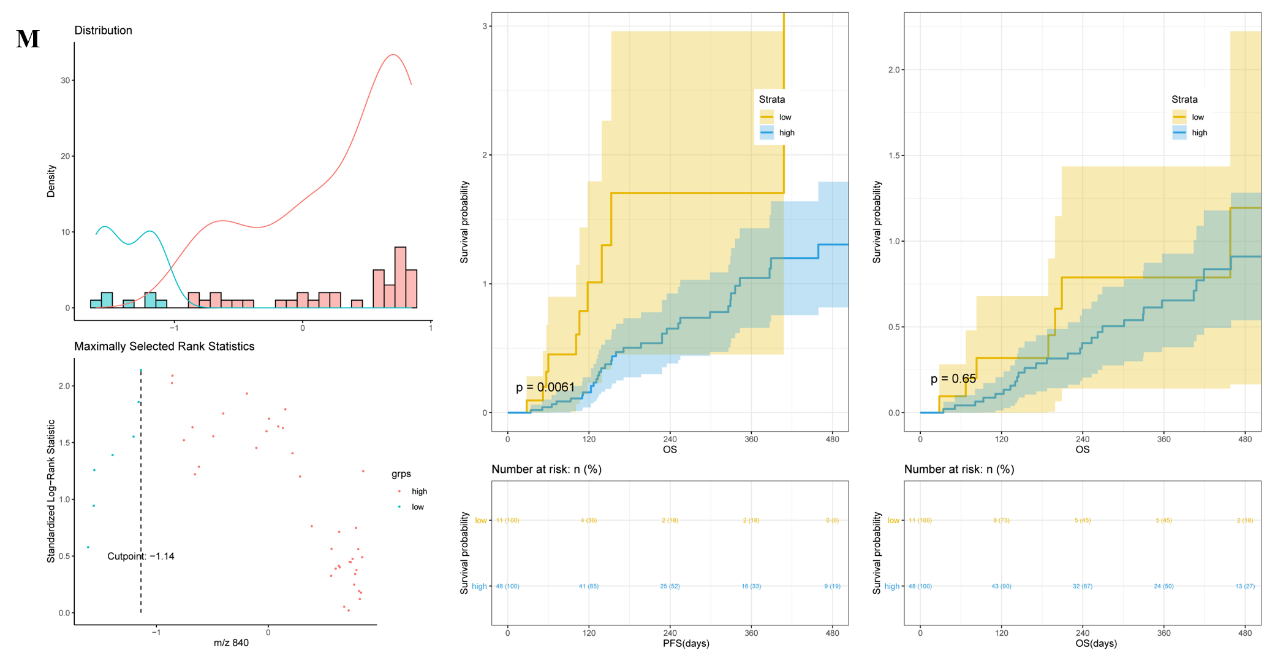


Figure S6. The cut-off values of metabolites were determined and divided into high and low metabolites groups. Plot of standardized log-rank statistics of metabolites (left panels), the PFS curve (middle panels) and OS curve according to the cut point of metabolites (right panels).

A. The cut-off values and Kaplan–Meier plot of *m/z* 386 [DG(O-18:0/2:0/0:0)+NH_4_-H_2_O]^+^,

B. The cut-off values and Kaplan–Meier plot of *m/z* 496 [LysoPC (16:0)+H] ^+^,

C. The cut-off values and Kaplan–Meier plot of *m/z* 620 [PA (30:0)+ NH_4_-H_2_O] ^+^,

D. The cut-off values and Kaplan–Meier plot of *m/z* 673 [DG (11M3/9M5/0:0)+H] ^+^,

E. The cut-off values and Kaplan–Meier plot of *m/z* 674 [PE-NMe (30:2)+H] ^+^,

F. The cut-off values and Kaplan–Meier plot of *m/z* 690 [DG (11D3/9D3/0:0) + NH_4_]^+^,

G. The cut-off values and Kaplan–Meier plot of *m/z* 706 [DG (42:10) + NH_4_] ^+^,

H. The cut-off values and Kaplan–Meier plot of *m/z* 707 [PE (32:1) + NH_4_]^+^,

I. The cut-off values and Kaplan–Meier plot of *m/z* 710 [PA (37:4) + NH_4_-H_2_O] ^+^,

J. The cut-off values and Kaplan–Meier plot of *m/z* 753 [PG (36:4)+H-H_2_O] ^+^,

K. The cut-off values and Kaplan–Meier plot of *m/z* 757 [PE (38:9)+ NH_4_-H_2_O] ^+^

L. The cut-off values and Kaplan–Meier plot of *m/z* 797 [PG (38:5)+H] ^+^,

M. The cut-off values and Kaplan–Meier plot of *m/z* 840 [PG (40:6)+H] ^+^

**Table S1** Identified metabolites from plasma samples

| No. | Compound name | Observed *m/z* | Formula | Adduct | Theoretical *m/z* | Error (Da) | VIP |
| --- | --- | --- | --- | --- | --- | --- | --- |
| 1 | Pyruvatoxime | 104.0165 | C_3_H_5_NO_3_ | [M+H]^+^ | 104.0528 | 0.0363 | 3.0 |
| 2 | 4-Ipomeanol | 169.0884 | C_9_H_12_O_3_ | [M+H]^+^ | 169.0859 | 0.0025 | 1.9 |
| 3 | Amifostine | 215.0184 | C_5_H_15_N_2_O_3_PS | [M+H]^+^ | 215.0614 | 0.043 | 2.9 |
| 4 | DG (O-18:0/2:0/0:0) | 386.3406 | C_23_H_46_O_4_ | [M+NH_4_-H_2_O]^+^ | 386.3623 | 0.0217 | 2.1 |
| 5 | LysoPC (16:0) | 496.3422 | C_24_H_50_NO_7_P | [M+H]^+^ | 496.3398 | 0.0024 | 1.6 |
| 6 | 3b,16a,21b,22a)-12-Oleanene-3,16,21,23,28-pentol-22-angeloyloxy-23-al | 604.4204 | C_35_H_54_O_7_ | [M+NH_4_]^+^ | 604.4208 | 0.0004 | 2.2 |
| 7 | PA (30:0) | 620.4405 | C_33_H_65_O_8_P | [M+NH_4_-H_2_O]^+^ | 620.4644 | 0.0239 | 2.1 |
| 8 | Saponin H | 650.3941 | C_36_H_58_O_10_ | [M+NH_4_-H_2_O]^+^ | 650.4257 | 0.0316 | 2.2 |
| 9 | DG (11M3/9M5/0:0) | 673.5024 | C_41_H_68_O_7_ | [M+H]^+^ | 673.5038 | 0.0014 | 2.2 |
| 10 | PE-NMe (30:2) | 674.46 | C_36_H_68_NO_8_P | [M+H]^+^ | 674.4755 | 0.0155 | 1.8 |
| 11 | DG (11D3/9D3/0:0) | 690.5242 | C_41_H_68_O_7_ | [M+NH_4_]^+^ | 690.5303 | 0.0061 | 2.5 |
| 12 | DG (42:10) | 706.5234 | C_45_H_68_O_5_ | [M+NH_4_]^+^ | 706.5405 | 0.0171 | 2.6 |
| 13 | PE (32:1) | 707.5269 | C_37_H_72_NO_8_P | [M+NH_4_]^+^ | 707.5334 | 0.0065 | 2.9 |
| 14 | PA (37:4) | 710.4937 | C_40_H_71_O_8_P | [M+NH_4_-H_2_O]^+^ | 710.5114 | 0.0177 | 1.7 |
| 15 | PE (36:4) | 740.5445 | C_41_H_74_NO_8_P | [M+H]^+^ | 740.5225 | 0.022 | 2.3 |
| 16 | PG (36:4) | 753.5169 | C_42_H_75_O_10_P | [M+H-H_2_O]^+^ | 753.5071 | 0.0098 | 1.9 |
| 17 | PE (38:9) | 757.4687 | C_43_H_68_NO_8_P | [M+NH_4_-H_2_O]^+^ | 757.491 | 0.0223 | 2.2 |
| 18 | PG (38:5) | 797.5432 | C_44_H_77_O_10_P | [M+H]^+^ | 797.5327 | 0.0105 | 2.0 |
| 19 | PG (40:6) | 840.5657 | C_46_H_79_O_10_P | [M+H]^+^ | 840.5749 | 0.0092 | 2.2 |
